# The effects of marine traffic on the behaviour of Black Sea harbour porpoises (*Phocoena phocoena relicta*) within the Istanbul Strait, Turkey

**DOI:** 10.1371/journal.pone.0172970

**Published:** 2017-03-15

**Authors:** Aylin Akkaya Bas, Fredrik Christiansen, Ayaka Amaha Öztürk, Bayram Öztürk, Caley McIntosh

**Affiliations:** 1 Faculty of Fisheries, Istanbul University, Beyazit, Istanbul, Turkey; 2 Turkish Marine Research Foundation, Beykoz, Istanbul, Turkey; 3 Marine Mammals Research Association, Antalya, Turkey; 4 Cetacean Research Unit, School of Veterinary and Life Sciences, Murdoch University, Murdoch, Western Australia, 6150 Australia; Institute of Deep-sea Science and Engineering, Chinese Academy of Sciences, CHINA

## Abstract

Marine traffic is threatening cetaceans on a local and global scale. The Istanbul Strait is one of the busiest waterways, with up to 2,500 vessels present daily. This is the first study to assess the magnitude of short- and long-term behavioural changes of the endangered Black Sea harbour porpoises (*Phocoena phocoena relicta*) in the presence of marine vessels within the Istanbul Strait. Markov chains were used to investigate the effect of vessel presence on the transition probability between behavioural states (diving, surface-feeding and travelling), and to quantify the effect on the behavioural budget and bout length (duration of time spent in a given state) of porpoises. Further, the changes on swimming directions of porpoises in relation to vessel speed and distance was investigated using generalized linear models. In vessel presence, porpoises were less likely to remain in a given behavioural state and instead more likely to switch to another state. Because of this, the bout length of all three behavioural states decreased significantly in the presence of vessels. The vessel effect was sufficiently large to alter the behavioural budget, with surface-feeding decreasing significantly in the presence of vessels. However, when taking into account the proportion of time that porpoises were exposed to vessels (i.e. 50%), the measured effect size was not large enough to significantly alter the animals’ cumulative (diurnal) behavioural budget. Additionally, vessel speed and distance had a significant effect on the probability of porpoises showing a response in their swimming directions. The southern and middle sections of the Istanbul Strait, which have the heaviest marine traffic pressure, had the lowest porpoise sightings throughout the year. Conversely, northern sections that were exposed to a lesser degree of marine traffic hold the highest porpoise sightings. The effect shown in this study in combination with increasing human impacts within the northern sections should be considered carefully and species-specific conservation actions, including establishment of protected areas, should be put in place to prevent the long-term consequences of marine traffic on the Black Sea harbour porpoise population.

## Introduction

The Black Sea harbour porpoise (*Phocoena phocoena relicta*) is recognised as a subspecies of the harbour porpoise (*P*. *phocoena*). The species is commonly found in shallow waters (0–200 m deep) over the continental shelf around the entire perimeter of the Black Sea, although they may also occur further offshore within deeper waters [1]. The Black Sea harbour porpoise is completely isolated from the nearest *P*. *phocoena* population in the North Eastern Atlantic [2], and is endemic to the Black Sea and neighbouring waters. Their full range extends over the Black Sea, Azov Sea, Kerch Strait, Turkish Straits System, and Northern Aegean Sea [2]. According to the IUCN Red List of Threatened Species [1], the Black Sea subspecies is at much greater risk of decline and thus classified as endangered under A1d and 4c,d,e categories. Although the actual population size present within the Black Sea is unknown, the current population size is believed to be at least several thousand animals [1, 3].

Up until 1983, the main threat to the Black Sea harbour porpoises was unregulated and uncontrolled harvesting [4]. At present, incidental mortality due to fishing nets represents the most serious threat, followed by overfishing, habitat loss, and chemical pollution [5]. A mass mortality event in 1982 in the Azov Sea due to gas explosion, along with two more mortality events in 1989 and 1990, together with habitat degradation and a decline in the prey availability (starting from the late 1980s), have also contributed to their listing as endangered [1, 3]. Furthermore, vessel-cetacean collisions have been frequently reported throughout the Mediterranean Sea [6], with a considerable skew towards mysticeti species [7]. However, small cetaceans, such as harbour porpoises, are also at risk from vessel collisions and have been previously reported with wounds from fatal boat strikes [8]. Even though there are no reported cases of vessel-porpoise collisions within the Istanbul Strait, the risk of collision within this high vessel density area should not be ignored [7].

Intense and increased use of coastal and maritime areas by humans has undoubtedly created environmental pressure within the Turkish Straits System and Black Sea [9]. Anthropogenic impacts to marine life are particularly severe due to the semi-enclosed nature of the area [10,11]. Potential effects of marine traffic on cetaceans in the Istanbul Strait and Black Sea have been cited by a few studies [5, 12, 13]. These studies have stated that high marine traffic can disrupt cetaceans within the Istanbul Strait, Black Sea and Azov Sea; however, no further research has since been conducted to investigate the impact of marine traffic on cetaceans, including Black Sea harbour porpoises.

Multiple studies have reported both short-term and long-term behavioural changes for several species of cetaceans in response to increasing marine vessel pressure [14–27]. Short-term changes can manifest themselves as behavioural changes, including variations in vocalisation, an increase in dive intervals, vertical and horizontal avoidance, and an increase in swimming speed and a decrease in resting behaviour [25]. Long-term changes can involve population decline and/or abandonment of an affected habitat [28, 29]. Lusseau [30] noted that behavioural budgets of a population can be directly related to their energy budget. Thus, changes in an animal’s behavioural budget over extended periods of time can result in energy depletion for that individual [30]. If a sufficiently large proportion of the population is affected, such energetic effects can eventually lead to long-term negative effects on the population [30, 31].

The Istanbul Strait (41°13'–41°00' N, 29°08'–28°59' E) is situated between the Black Sea and the Mediterranean Sea. Although the Strait is an important habitat for marine life, it also renders important economic value for commuting, shipping, fishing, and recreational activities. Commercial cargo vessels, ferries, sea buses, speed boats, and industrial and artisanal fisheries are common within this area, resulting in dense marine traffic. When the Montreux Agreement was signed in 1936, the number of commercial vessels passing through the Istanbul Strait was approximately 4,500 per year, in comparison to the 46,000 vessels passing through the Strait annually today [32]. Official statistics have reported that, on average, 130 commercial cargo vessels and 2,500 domestic vessels pass through the Strait every day [32–34].

Impact studies in other geographical locations suggest that minimising boat-cetacean interactions is an important element in management of anthropogenic impacts on cetaceans. Thus, decreasing boat pressure is vital for the protection of a species, specifically in their critical habitats [35, 36]. However, a sustainable management strategy requires an in-depth knowledge and understanding of the targeted species and its vulnerability to marine traffic. In order to establish this understanding, a behavioural impact study of marine traffic on the target species is needed [15]. In this study, we investigated the effect of vessel traffic on the behaviour of Black Sea harbour porpoises in the Istanbul Strait. We first compare the behavioural transition probabilities of porpoises during impact (vessels present) and control (no vessel present) situations using Markov chain analysis, and the effect of vessel traffic on the behavioural budget and bout duration of porpoises. Further, we tested the effect of vessel speed and distance on the probability of changes in swimming direction of porpoises to better understand what factors might be driving their behavioural responses.

## Materials and methods

### Data collection

#### Survey platforms

**Fig 1:**
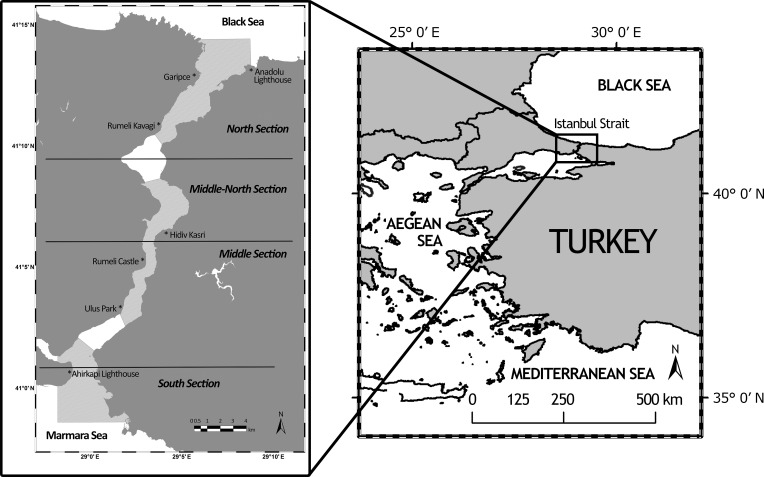
Sections and observation stations in the Istanbul Strait, Turkey (coverage of land-based survey is shown in light gray).

Porpoise and vessel data were collected by weekly systematic land and boat surveys between September 2011 and September 2013. Land surveys were conducted from seven theodolite stations within four different sections of the Istanbul Strait (Fig 1). The permission to use Ahırkapı Lighthouse has been issued by Directorate General of Coastal Safety, while General Directorate of Cultural Heritage and Museums has issued the permissions for Rumeli Castle and Hidiv Kasrı. For the rest of the observation stations, no specific permission was required as they were accessible to the public. Each station was visited on at least two different days each month with a daily average of 5 hours. Theodolite stations were selected along the coastline at least 30m above the sea level. Reference points and the exact positioning of the theodolite placement were kept constant throughout the study. The location and behaviour of harbour porpoises and marine vessels were recorded using a theodolite linked to the tracking software Pythagoras v. 1.2 to transform theodolite readings into geographic positions. When vessels and cetaceans were present together, coordinate points were recorded for the vessels and the focal group alternately.

Boat-based observations covered the entire strait and were conducted on three different days per month, independent of land survey days. A 16m gullet boat with a 185 horsepower engine was utilised throughout the surveys. The boat was operated along pre-determined transect lines at a speed of around 4knots. Focal porpoise groups were typically followed at a distance of 50 to 400m from the side or rear. If an individual happened to approach the research vessel closer, speed was gradually reduced until ‘idle speed’ was reached, and any sudden movements of the vessel were avoided in order to minimise the impact of the researchers on the animals. Any changes on the swimming direction of the focal group due to the approach and presence of the research vessel were recorded. All sightings and effort data, as well as environmental and survey conditions, were recorded during both land-based and boat-based surveys.

#### Behavioural sampling

Group focal follow was conducted in order to determine the predominant behaviour of the harbour porpoises, i.e. the behavioural state in which >50% of the porpoises in a group are engaged in. A group was defined as individuals engaging in similar behaviors, with close-group cohesion (less than 50m). The behavioural state of the focal group was sampled every 3 minutes using scan sampling methods. Behavioural states were identified as ‘travelling’, ‘diving’, ‘surface- feeding’, ‘milling’, ‘resting’, and ‘socialising’ (Table 1) [15, 25, 37, 38, 39]. Later, milling, resting and socializing behaviour were discarded from the analysis due to their small sample size.

**Table 1:** Definition of each behavioural state of porpoises used in this study.

Behavioural State	Definition
**Travelling (TR)**	Porpoises engage in directional movement, and make noticeable headway with constant speed. Dive intervals are relatively short (≤15 sec).
**Diving (DV)**	Coordinated, steep dives are seen in various directions. No obvious, steady movements are recorded. Possibly linked to foraging activity.
**Surface-feeding (SU-FE)**	Porpoises chase fish, majority of the behaviour takes place close to the sea surface with rapid directional changes. Prey often observed at the sea surface, along with ripples.
**Milling (MI)**	Non-directional movement and frequent changes in bearing. Although the group movement varies, group cohesion stays similar.
**Resting (RE)**	Porpoises observed within a tight group (≤5m) with synchronous and steady movements and swimming speed is low (≤1knot) with short dive intervals (≤15 sec).
**Socializing (SOC)**	Diverse interactive events (i.e. body contacts, tail slaps, synchronise full leaps). Aerial behavioural events are frequently observed with varied dive intervals.

Sampling of sequential behavioural states was dependent upon the conspicuity of the group. To illustrate, if the group was not visible in the 3 minutes after the original sampling time, their next sighting was recorded and the sampling interval restarted with the time of the latter sighting. If the focal group was out of sight for more than 20 minutes, the next sighting was declared as a new group. In the case of multiple groups present at the same time, only the first sighted group behaviour was noted and the rest of the groups were ignored.

Changes in swimming direction of porpoises in relation to the nearest vessel type were recorded for each behavioural sampling unit and categorised as either: (a) response–when porpoises swam away or towards a vessel, or; (b) no response–when porpoises kept a constant direction despite vessel presence.

#### Marine vessel sampling

Three separate marine vessel datasets were collected during the surveys, including (1) Marine vessel type and density: the number of marine vessels, according to type, was counted every 10 minutes in order to estimate the marine vessel density of each station. This type of data was collected only during land surveys, and separately from porpoise sightings. Vessels were divided into 9 different categories; HSB (high-speed boat), FB (fishing boat, <10m in length), FV (fishing vessel, >10m in length, usually equipped with a sonar system), RB (research boat), FE (ferry), SB (sea bus), SCS (small commercial cargo, <200m in length), BCS (big commercial cargo, >200m in length) and IDLE (idle speed of all the above vessels). (2) Nearest marine vessel type to the focal group: The nearest vessel to the porpoises was recorded for each behavioural sample in order to assess the possible impact of the nearest vessels on swimming directional changes. The accurate distance between the nearest vessels and the focal group was measured using Pythagoras linked to the theodolite. The nearest vessel data from boat surveys was discarded, as the distance was estimation. Marine vessels were placed within one of three speed categories: (a) slow vessels–idle speed up to 3knots; (b) medium vessels– 3knots to 9knots, and; (c) fast vessels– 9knots and upwards. (3) The number of vessels within 400m and 1,000m of the porpoises, were counted for each behavioural sampling unit during land surveys.

#### Behavioural transitions

The number of transitions between different behavioural states were used to create two-way contingency tables between preceding (the behavioural state recorded at time *t* minutes) and succeeding (the behavioural state recorded at time *t + 3* minutes) states during control and impact situations [15]. If no vessels were recorded for a continuous period of 15 minutes between the preceding (P) and succeeding (F) behaviour, the transition was added to the control table. If marine vessels were present within 400m of the focal group, the transition between preceding and succeeding was added to the impact table [15]. Only focal follows containing a minimum of three transitions, during both land and boat surveys, were included in analyses. Although the control chain represents no marine vessel presence within the 400m zone, it was highly likely that vessels were, in fact, present beyond this distance.

### Statistical analysis

#### Sightings

To understand the effect of seasons, sections and survey type on porpoise sightings, a Poisson regression was fitted to the data. However, due to the over dispersion of the data, negative binomial with loglink was the selected model type. While the count data of porpoise sightings was used as the response variable, seasons (spring, summer, autumn, winter), sections (south, middle, middle-north and north) and survey types (land and boat surveys) were used as explanatory variables, and the survey effort in days was selected as an offset (S1 Dataset).

#### Markov chain and model selection on behavioural transitions

Time-discrete Markov Chain analyses are widely applied technique to quantify the one-way dependence of an event on the preceding event which allows the possible effect of any factor on the dependence of the events to be assessed [15, 25, 30, 39–42]. Therefore, a contingency table (four seasons vs. four sections vs. two marine vessel states vs. three preceding behaviours vs. three succeeding behaviours) was created by merging the control and impact chain for all seasons and sections. Marine vessel (M), season (S) and section (L to avoid abbreviation confusion) effects on the first order behavioural transitions from preceding (P) to succeeding (F to avoid abbreviation confusion) were assessed using a log-linear analysis, as described in detail by Lusseau [15, 30] and Lusseau et al. [41]. While the model’s null hypothesis stated that succeeding behaviours were independent of marine vessels, seasons and sections, given the preceding behaviour, coded as *PF*, *MSL*, the fully saturated model (coded as MSLPF) stated that succeeding behaviours were dependent on all possible interactions of seasons, sections and vessels. Starting with the null model, each factor was added to the initial model one by one until the saturated model was reached. The significance of each added factor was tested by comparing the goodness-of-fit of the initial model against its later model [15]. The best fitting model on the explanation of behavioural transitions was selected based on their Akaike Information Criterion (AIC) [15].

#### Behavioural transition probabilities

Behavioural transition probability matrices were developed by calculating transition probabilities (from preceding to succeeding behavioural state) for both the impact and control chain [15]:

$$p_{ij}=\frac{a_{ij}}{\sum_{j=1}^{3}a_{ij}},\sum p_{ij}=1$$

where *p* is the transition probability between preceding behavioural state *i* and the succeeding behavioural state *j* (*i* and *j* range from 1 to 3 due to the 3 behavioural states), and *aij* is the number of transitions observed from behavioural state *i* to *j* [15]. To test the effect of vessel interaction on the transition probability of porpoises, impact and control chains were compared using a chi-square test where the observed number of transitions corresponded to the impact contingency table and the expected number of transitions corresponded to the control contingency table [15, 42]. In addition, each control transition was compared to its corresponding impact transition (3*3 = 9 in total), using a 2-sample test for equality of proportions with continuity correction (S1 File) [15, 42, 43].

#### Behavioural budgets

To investigate the effect of vessel presence on the behavioural budget (the proportion of time spent in different behavioural states), left eigenvectors of the dominant eigenvalues of the transition matrices were calculated both for control and impact matrices [15, 30]. Due to the ergodic nature of the Markov chains, initial behavioural states can converge toward a stationary behavioural distribution, which is proportional to left eigenvectors and corresponds to the behavioural budget of the population [15, 30]. The differences between the control and impact behavioural budgets were tested using a chi-square test [15, 42, 43]. Each behavioural state within the control behavioural budget was compared to the corresponding behavioural state within the impact behavioural budget by using a 2-sample test for equality of proportions with continuity correction. The 95% confidence intervals were calculated for the estimated proportion of time spent within each behavioural state (S1 File) [15, 42].

#### Bout lengths

Average bout lengths (the duration of time spent in a given state) of each behavioural states $\bar{t_{ii}}$ were estimated for both the control and impact chain, as described by Lusseau [15, 30];

$$\bar{t_{ii}}=\frac{1}{1-p_{\begin{array}{l} ii \end{array}}}$$

with a standard error of $\text{SE}=\sqrt{\frac{p_{ii}\times (1-p_{ii})}{n_{i}}}$

where *n_i_* is the number of samples with *i* as preceding behaviour. Later, bout lenghts were compared between the control and impact situation using a Student's t-test (S1 File).

#### Cumulative behavioural budgets

A cumulative behavioural budget is able to account for the time porpoises spend in both the control and impact behavioural budgets. By artificially varying the proportion of time that porpoises spend with vessels per day from 0 to 100%, it is possible to see at what level of vessel intensity the cumulative behavioural budget becomes significantly different from the control budget, given the observed effect size (the estimated behavioural budgets) and assuming that such effect size does not vary with the daytime exposure rate [30, 39, 42]. The effect of vessels on the daytime behavioural budget of porpoises can be investigated by comparing the cumulative behavioural budget with the control budget. The cumulative behavioural budget was calculated following Lusseau [30] and Christiansen et al. [42]:

Cumulative budget=(a×impact budget)+(b×control budget)

where *a* is representative of the proportion of time porpoises spend with a marine vessel, and *b* is the remaining proportion of time (1-a) spent without vessels. The difference between the cumulative behavioural budget and the control budget was tested with a chi-square test and 2-sample test for equality of proportions with continuity correction for each behavioural state (S1 File) [42, 43].

#### Changes in swimming direction

To investigate which vessel-related variable affects the directional response (response vs. no response) of porpoises, a generalized linear model (GLM) with a binomial distribution (response as a binary variable) and a logit link function were fitted to the data collected during land surveys. The covariates investigated were distance to the nearest vessel, the speed category of the nearest vessel (slow, medium and fast), the number of vessels within 400m and the number of vessels within 1,000m of the porpoises. To account for temporal auto-correlation within follows, and uneven sample sizes between follows, only the first two data point from each follow was used in the analyses. Collinearity (high correlation) between the explanatory variables in the final model was investigated by estimating the variance inflation factor (VIF), with an upper threshold value of three indicating collinearity. Overdispersion was tested by dividing the residual deviance by the residual degrees of freedom, with a ratio value (dispersion parameter, φ) above one indicating overdispersion (the mean of the variance is larger than the mean). The best fitting model was selected using AIC (S2 File).

The level of significance for all of the above analyses was selected under 0.05 thresholds with a 95% of confidence interval. Statistical analyses were performed using the statistical software SPSS 20 and R 3.1.1 [44].

## Results

### Sightings

A total of 365 days (1928 hours) were spent searching for porpoises throughout the Istanbul Strait. Of these, 57 days were spent at sea and 308 days on land. In total, 477 focal group follows were undertaken over 114 days (70.6 hours), with 29 days (12 hours) being conducted from the research vessel and 85 days (58.6 hours) from land. A group follow ranged from one sampling unit (3 minutes) to 31 unit (93 min), with an average of 4.23 sampling units. Over the course of the study, the behavioural data of the porpoises was recorded within 1,403 cases of scan samples, which later corresponded to 658 behavioural transitions. Of these transitions, 364 were classified under the control chain and 294 were classified under the impact chain (S1 and S2 Tables).

**Fig 2:**
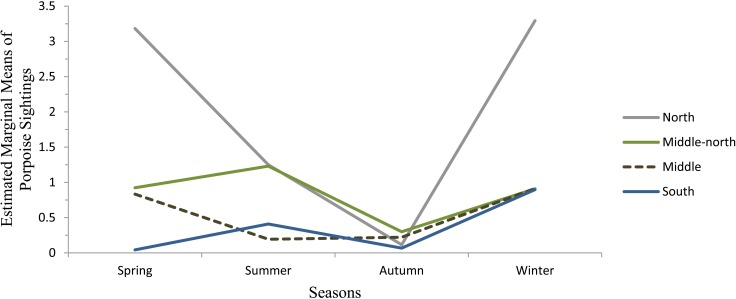
Number of porpoise sightings as a function of season and section of the Istanbul Strait.

Regarding the changes on the porpoise sightings based on the seasons, sections and survey types, survey type had no significant effect (χ^2^ = 0, df = 1, p = 0.99). However season (χ^2^ = 22.64, df = 3, p<0.0001) and section (χ^2^ = 11.316, df = 3, p = 0.02) showed a significant effect on the sightings. Sightings within the north section were 3.67 times higher than the south section. The north section had the highest sightings during all seasons (winter = 3.29; spring = 3.18; and summer = 1.25 groups per survey) except autumn (0.12 groups per survey). In autumn, sightings across all sections were below 0.5 groups per survey (Table 2, Fig 2). The south and middle sections held the lowest sightings all year round, with an average of 0.35 and 0.54 group per survey, respectivelly.

**Table 2:** Porpoise sightings per seasons and sections within the Istanbul Strait.

Season	Section	Encounter in days	Total group number.	Survey effort in days
**SPRING**	*South*	1	1	23
	*Middle*	7	20	24
	*Middle-north*	4	12	13
	*North*	22	140	44
**SUMMER**	*South*	7	9	22
	*Middle*	6	6	31
	*Middle-north*	5	16	13
	*North*	21	65	52
**AUTUMN**	*South*	2	2	30
	*Middle*	3	6	27
	*Middle-north*	1	3	10
	*North*	5	5	43
**WINTER**	*South*	8	26	29
	*Middle*	8	21	23
	*Middle-north*	4	10	11
	*North*	20	135	41

**Fig 3:**
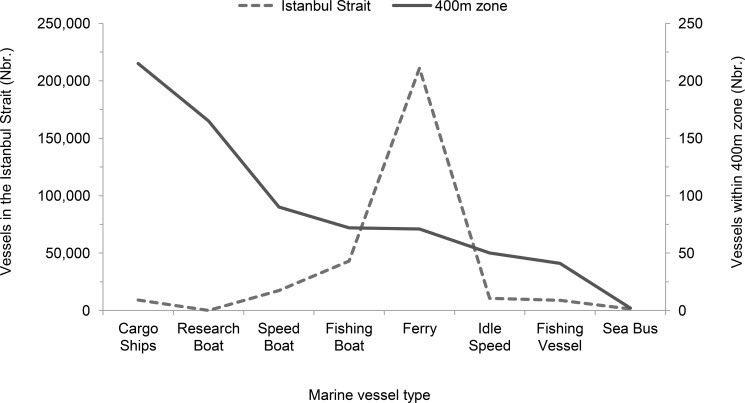
Overall vessel number for each type that was present within 400m and their overall count during the study within the Istanbul Strait (The overall count of each marine vessel type was independent of porpoise presence).

Porpoises spent 49.6% of overall observation time (boat+land surveys) within the 400m radius of marine vessels in the Istanbul Strait. Up to 56 vessels were recorded within 1 km of a porpoise group, with a mean of 1.87 vessels **±**0.09 SE. Regardless of porpoise presence, the 10 minute sampling interval of marine vessel data revealed an estimation of 301,247 marine vessels present throughout the study period (between 2011 and 2013). The highest marine vessel density (210,963 vessels or 70% of the total traffic) was recorded within the middle section, followed by the south section (38,263 vessels or 13% of the total traffic), the north section (35,482 vessels or 12% of the total traffic) and the middle-north section (16,483 vessels or 5% of the total traffic). Ferries were the most dominant vessel class in all sections, except the north section where fishing boats were dominant vessel class. Ferries were responsible for 70% (211,444 vessels) of the total marine traffic (Fig 3). However, the majority of porpoise-vessel encounters were recorded with cargo ships, along with the research boat (Fig 3).

### Markov chain and model selection on behavioural transitions

**Fig 4:**
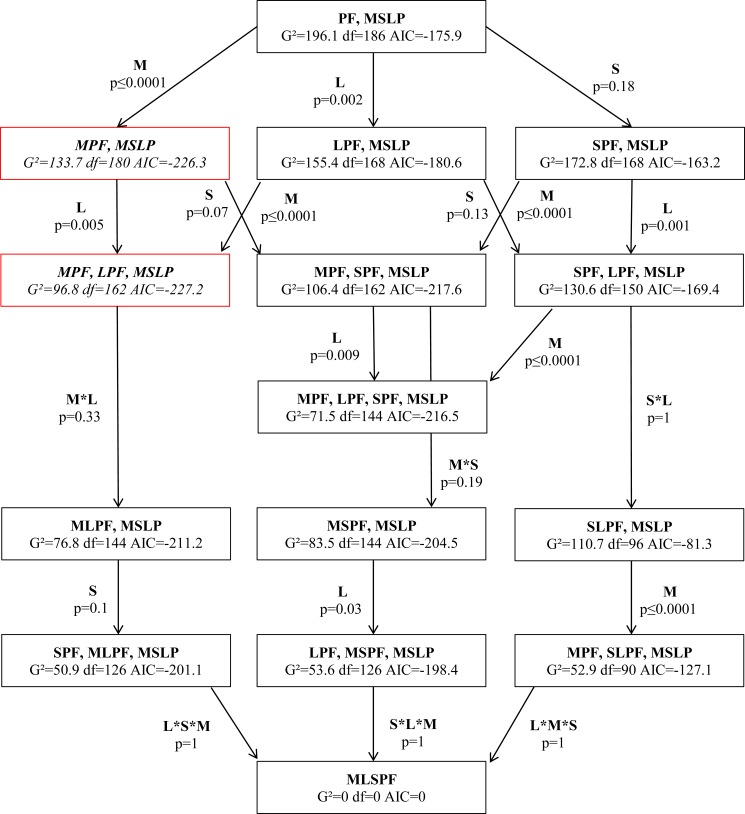
Model testing for marine vessel (M) presence at 400m, season (S) and section (L) effects on behavioural transitions from preceding (P) to succeeding (F) using log-linear analyses. Models and their respective goodness-of-fit G^2^ statistics, degrees of freedom, and AIC values are shown in the boxes. Red outlined boxes are the best fitted models. Arrows represent the flow between the models. The added factors and their significance are shown along the arrows. Asterisks indicate an interaction term between variables (adapted from Lusseau 2003).

Log-linear analysis showed that "Marine vessel (MPF, MSLP)" and "marine vessel and section (MPF, LPF, MSLP)" models were the most supported model based on their lowest AIC values on the variance of behavioural transitions (Fig 4). Neither the saturated model (which considers all of the interactions between vessel, season and section (MLSPF)) nor the null model (which disregards all of the factors (PF)) provided a significant change on the behavioural transitions. Starting with the null model (PF,MSLP), each factor (vessel, season and section) and their interaction term was added to the following model until the saturated model was reached (Fig 4). While vessel and section had a significant effect on the behavioural transitions in each model, season factor was not significant in the explanation of behavioural transitions (Fig 4).

### Behavioural transition probabilities

**Fig 5:**
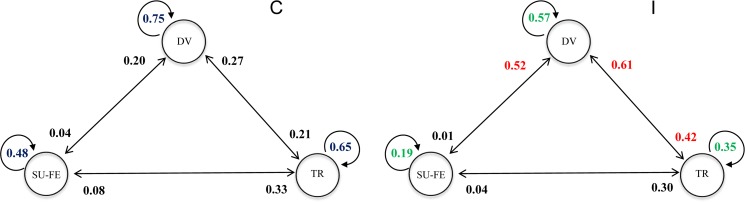
Transition matrices for control chain (C) and impact chain (I). Behavioural states were diving (DV), travelling (TR), and surface-feeding (SU-FE). The numbers represents probabilities. While the green text shows significant decline in the presence of vessels, the red text shows a significant increase.

**Fig 6:**
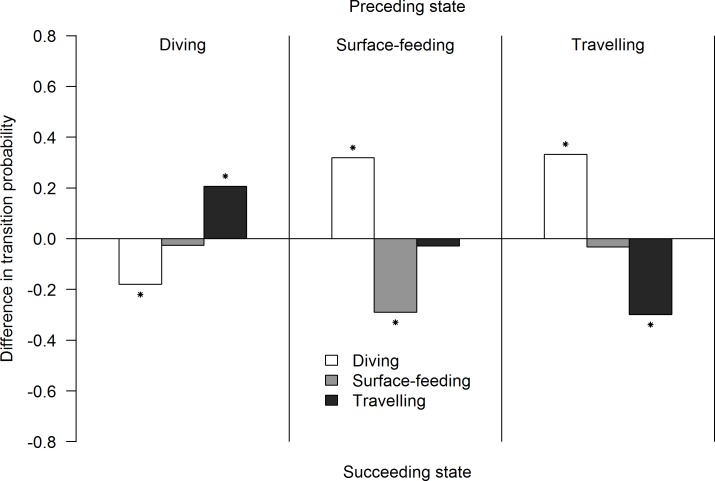
Differences in behavioural transitions between the control and impact chain (p_ij(impact)-_p_ij(control)_). The vertical line separates each preceding behavioural state, while the succeeding behavioural state is represented by bars. Asterisks indicate significant behavioural transitions (p<0.05).

The Markov chain analysis showed that behavioural transitions significantly changed in the presence of marine vessels (Goodness-of-fit test, χ^2^ = 158.09, df = 4, p<0.0001). Vessel presence significantly affected six of nine behavioural transitions (Fig 5). Three of the transitions, Diving to Diving (Z-test = 9.19, p = 0.002, control = 75% 69–80 CI95%, impact 57% 51–63 CI95%,), Travelling to Travelling (Z-test = 26.62, p<0.0001, control = 65% 60–70 CI95%, impact = 35% 30–41 CI95%) and Surface-feeding to Surface-feeding (Z-test = 4.7, p = 0.03, control = 48% 42–53 CI95%, impact = 19% 14–24 CI95%), significantly decreased in the presence of vessels. On the other hand, the probability of changing from Diving to Travelling (Z-test = 12.76, p<0.0001, control = 21% 17–26 CI95%, impact = 42% 36–48 CI95%), Surface-feeding to Diving (Z-test = 6.04, p = 0.014, control = 20% 16–25 CI95%, impact = 52% 50–58 CI95%) and Travelling to Diving (Z-test = 33.51, p< 0.0001, control = 27%, 23–32 CI95%, impact = 61% 55–66 CI95%) significantly increased (Fig 6).

### Behavioural budgets

**Fig 7:**
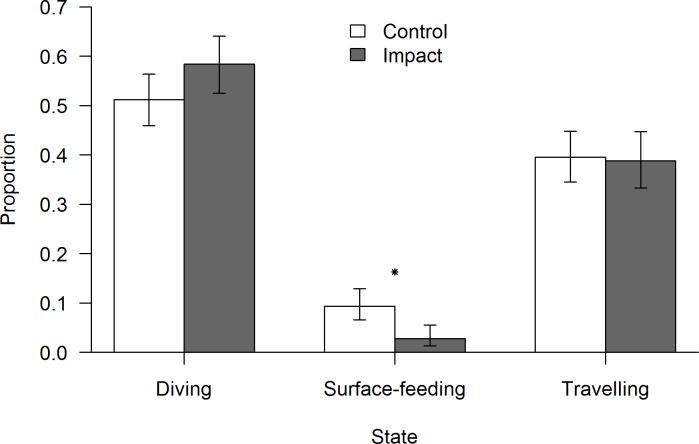
Behavioural budget of control and impact chain. Error bars represent 95% confidence intervals. An asterisk indicates significant differences between behavioural transitions (p<0.05).

In the absence of vessels, porpoises spent most of their time diving, followed by travelling and surface-feeding (Fig 7). The behavioural budget was significantly affected by the presence of vessels (Goodness of fit test, χ^2^ = 14.59, df = 2, p<0.0001). The proportion of surface-feeding was significantly lower in the impact budget (Z-test = 10.53, p = 0.001, control = 9%, impact = 2%). Nonetheless, the proportion of time spent diving (Z-test = 3.13, p = 0.07) and travelling (Z-test = 0.01, p = 0.9) did not differ between control and impact situations.

### Bout lengths

**Fig 8:**
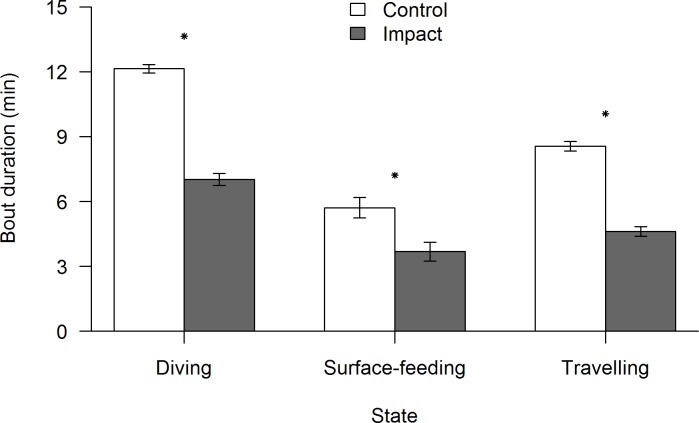
Bout lengths of each behavioural state during the control (white) and impact (gray) situations. Error bars represent 95% confidence intervals. Asterisks indicate significant behavioural transitions (p<0.05).

The average bout lengths (min.) of all three behavioural states showed a significant decline in the presence of vessel traffic (Fig 8). The diving bout length was reduced from 12.14±0.1 SE during control situations to 7.02±0.14 SE during impact situations (Student t-test = 30.512, df = 278, p<0.0001), while surface-feeding was also reduced from 5.17±0.24 SE to 3.68±0.22 (Student t-test = 5.94, df = 65, p<0.0001). Travelling bout length also significantly decreased from 8.55±0.12 during control situations to 4.62±0.11 during impact situations (Student t-test = 24.257, df = 309, p<0.0001). The diving and travelling bout durations were reduced by over 40%, along with a 36% decline in surface-feeding bout duration, during vessel presence.

### Cumulative behavioural budgets

**Fig 9:**
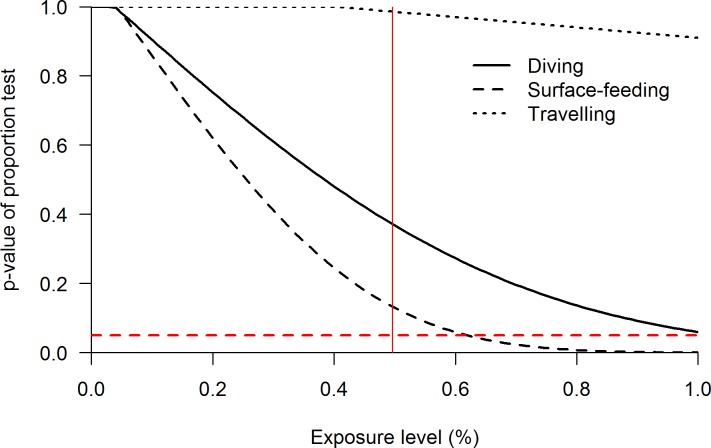
Effect of marine vessels on the cumulative behavioural budget of harbour porpoises during different levels of exposure. The y-axis represents the p-value of the difference between the cumulative behavioural budget and the control behavioural budget for the three behavioural states (see legend) at different vessel exposure levels. The dashed red line represent the statistical level of significance (p < 0.05). The solid red line indicates the current exposure level of porpoises to marine vessels in Istanbul Strait.

At the current vessel exposure level (49.6%), the cumulative behavioural budget was not significantly different from the control behavioural budget of porpoises (χ^**2**^ = 2.928, df = 2, p = 0.23). When effects were built linearly, only surface-feeding behaviour demonstrated significant differences when the vessel exposure reached up to 62% of daytime hours (Fig 9). The diving and travelling states did not show any significant difference between the cumulative and control budget, even if the porpoises were to spend all their daylight hours in the presence of marine vessels (Fig 9).

### Changes in swimming direction

**Fig 10:**
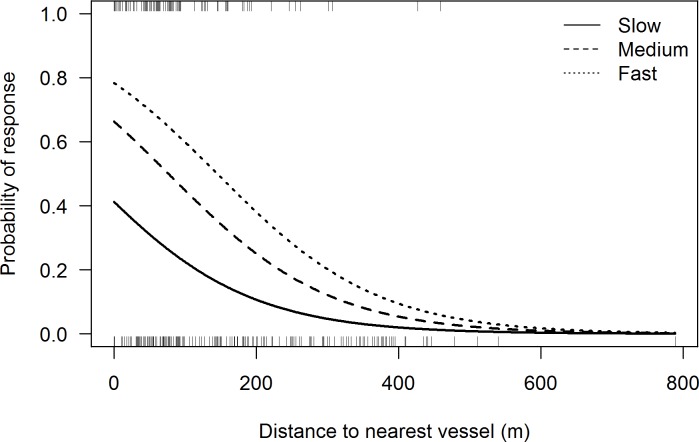
Probability of porpoises showing a response on their swimming direction towards vessels as a function of the distance to the nearest vessel for slow (solid line), medium (dashed line) and fast (dotted line) moving vessels. The lines represent the fitted values of the best fitting generalized linear model. The distribution of distance values for porpoises showing a response and no response are shown by the top and bottom rug plots, respectively. n = 305.

The best fitting GLM showed a significant effect of vessel distance (P<0.001, n = 305) and vessel speed (P<0.001, n = 305) on the response (directional changes) probability of porpoises. The number of vessels did not affect the response of porpoises towards vessels. The model explained 15.8% of the deviance (pseudo-R2) in the data. There was no collinearity between the explanatory variables in the best fitting model and no sign of overdispersion (φ = 1.06). The probability of porpoises showing directional responses to vessels decreased with the distance to the nearest vessel (logit scale: probability = -0.008, SE = 0.001) (Fig 10). The response was strongest for fast moving vessels (logit scale: probability = 1.253, SE = 0.404), compared to medium (logit scale: probability = 0.658, SE = 0.396) and slow moving vessels (logit scale: probability = -0.381, SE = 0.424) (Fig 10). The effect of distance did not differ between vessels from different speed categories, thus there was no interaction term in the model. At close distances (<50m), the response probability was around 40, 65 and 80% for slow, medium and fast moving vessels, respectively. As the distance to the nearest vessel increased, the probability of porpoises showing response decreased rapidly, to around 20, 40 and 60% at 100m and around 10, 30 and 40% at 200m, respectively (Fig 10). Beyond 400m, the response probability of porpoises was less than 10%, irrespective of the speed of the vessel (Fig 10) (S2 Dataset).

## Discussion

Assessing the effects of marine vessels on cetaceans have been the focus of many studies over the past two decades, in response to the global increase of marine traffic [5, 13–16, 21, 26–28, 30, 39, 45, 46]. Although various studies have focused on the effect of whale and dolphin watching tourism on cetaceans [31, 37, 40, 42, 47–49], fewer studies have discussed local marine traffic and international maritime impact on harbour porpoises [50–53]. The current study revealed that Black Sea harbour porpoises spend half of their daylight time within the 400 m range of marine vessels throughout the Istanbul Strait, and that marine traffic induces significant changes not only on swimming direction but also on behavioural transitions. In the close proximity of high speed vessels (<50m), porpoises changed their swimming direction up to 80% of the observations, yet this percentage dropped to 10% when vessel distance was over 400m. Our results on distance-response relationship are in line with previous studies [13, 54–58]. Further, vessel speed might lead to injuries, which is clear that the severity of injuries caused by an impact is likely to increase with vessel speed [59].

The average time porpoises spent in a behavioural state dropped for all the behaviours in the presence of vessels. Porpoises also had a reduced probability of remaining in the same behavioural state. They were more likely to shift their behaviour to diving in the vessel presence. The behavioural transitions were large enough to affect their behavioural budget, with surface-feeding showing a noticeable drop in the presence of vessels. However the relative time that they spent in each state overall did not change enough to alter the activity budget for diving and travelling.

It is well established that a decrease in surface-feeding behaviour can reduce energy intake and ultimately cause a long-term behavioural consequences as in reduce an animal’s health, survival and reproductive success [15, 37, 49, 54]. Even though a significant decrease of surface feeding in the budget raises concerns, the current vessel exposure was not sufficiently large to alter the porpoises' cumulative behavioural budget. Concerning the reliability of our results, all behavioural transitions occurred at least five times, with the exception of Diving to Surface-feeding, which only occurred once during impact situation. However because the transition probability between Diving and Surface-feeding was low both during control (0.04) and impact situations (0.01), it is unlikely that the low sample size during impact situations would have significantly influenced our results.

Despite the significant behavioural changes under vessel presence, the cumulative behavioural budget of porpoises wasn't significantly changed in the current exposure level (50%). The unchanged cumulative budgets might be linked to the area preference and/or behavioural adjustments of the animals. Porpoises might be compensating for reduced feeding opportunities during daytime by feeding more at night, when vessel activity is lower. A passive acoustic monitoring study in the middle-north section of the Strait detected the most click trains of delphinids and porpoises, indeed, at night [60], likely associated with foraging behaviour. However, further research into the nighttime behaviour throughout the Strait is needed to clarify the possible diurnal behavioural changes of porpoises.

Regarding the area avoidance behaviour, porpoises in the Istanbul Strait might be able to reduce their overall exposure to vessels, by spending more time in areas with lower and slower vessel traffic, represented by the northern sections in the strait. Our study provides evidence that porpoise sightings were indeed concentrated within the northern sections. The southern and middle sections had the lowest sightings throughout the year and have the heaviest marine traffic pressure, characterised by a disproportionally high number of high speed vessels. However, imperfect visual detectability of porpoises must be taken into account on the accuracy of area preferences. Seasonal area avoidance behaviour was also recorded, with a sharp decline in autumn sightings in the north and middle-north section.

Temporal area avoidance of dolphins during the high vessel activities was also documented in Australia [61]. Autumn in the Istanbul Strait is characterised by the pelagic fish migration and the start of the industrial fishing season. During this time, the north and middle-north sections was exposed to heavy fishing vessel pressure, with over 50 fishing vessels (purse seines) recorded simultaneously in 1km^2^. The south and middle sections are closed to fishing due to the risk of collision between fishing vessels and daily marine traffic. Although fishing vessel pressure was absent in the south and middle section, the lack of corresponding increase in autumn sightings rate indicates a probable lack of movement to these areas. It is possible that high fishing vessel density elicits a seasonal avoidance response from the entire Istanbul Strait, even at the expense of foraging during high prey density. Increased and consisting behavioural compensation on their area replacement and/or seasonal area avoidance, may lead to long-term energy depletion for affected individuals, thus potentially destabilising the entire population. Istanbul Strait serves as the only migration corridor for cetaceans between the Aegean Sea and the Black Sea [62]. Thus, increasing marine traffic might eventually act as a barrier between the Black Sea and the Aegean Sea.

Current study provided the first in-depth investigation of the vessel-porpoise interactions within the Istanbul Strait in order to implement effective and viable conservation actions for the Black Sea harbour porpoises. Despite it's one of the busiest waterway of the world, the Istanbul Strait lacks any kind of conservation and management measures for the porpoises that are listed as at risk. The proven behavioural transitions and avoidance responses of porpoises in response to the marine traffic, along with increasing human impacts on the north and middle-north sections, highlight the need for immediate conservation actions to mitigate the negative vessel impacts on the porpoise population. Lastly, regular surveys of the local population should be conducted to monitor the behavioural and biological changes under yearly varying marine traffic in the strait.

## Conclusion

Behavioural changes demonstrated by Black Sea harbour porpoises were related to marine vessel presence within the Istanbul Strait, and the effect on behavioural budgets is already significant. Surface-feeding was the only behaviour significantly affected by vessel presence within the budget. While slow speed vessels do not evoke a significant change on swimming directions, high speed vessels not only elicit a strong response, but could also lead to active area avoidance on a larger spatial scale. There is currently high marine traffic throughout the Istanbul Strait, with the same area pinpointed as one of the busiest international waterways, species-specific conservation measures and management strategies ought to be put in place immediately to avoid the long-term biological consequences. Such controls should consider vessel-free regions for the core zones of harbour porpoise habitats, enforced speed limits, marine vessel density limitations, and special channels specific for ferries within the Istanbul Strait.

## Supporting information

S1 File R codes for Markov Chain analysis. (R)

S2 File R codes for directional changes on porpoise swimming under the vessel speed, distance and density. (R)

S1 Table Control contingency table. (XLS)

S2 Table Impact contingency table. (XLS)

S1 Dataset Data on the porpoise sightings. (XLS)

S2 Dataset Original data on the swimming directional changes of porpoises. (XLS)

S3 Dataset Original data used during Markov Chain analysis and model selections. (XLS)

## References

[1] Birkun Jr AA, Frantzis A. Phocoena phocoena ssp. relicta. The IUCN Red List of Threatened Species [Internet]. 2008 [Downloaded on 27 January 2016]. Available from: e.T17030A6737111.

[2] Frantzis A, Gordon J, Hassidis G, Komnenou A. The enigma of harbour porpoise presence in the Mediterranean Sea. Marine Mammal Science. 2002;17(4):937–943.

[3] Reeves RR, Notarbartolo Di Sciara G. The status and distribution of cetaceans in the Black Sea and Mediterranean Sea. IUCN Centre for Mediterranean Cooperation, Malaga, Spain 2006.

[4] IWC. Annex L. Report of the Sub-committee on Small Cetaceans. Journal of Cetacean Research and Management. 2004;6(Suppl.):315–334.

[5] Birkun A Jr. Interaction between cetaceans and fisheries: Black Sea In: di Sciara G. Notarbartolo (ed.), Cetaceans of the Mediterranean and Black Seas: State of Knowledge and Conservation Strategies. ACCOBAMS Secretariat, Monaco 2002 pp. 98–107.

[6] Panigada S, Pesante G, Zanardelli M, Capoulade F, Gannier A, Weinrich MT. Mediterranean fin whales at risk from fatal ship strikes. Marine Pollution Bulletin. 2006;52:1287–1298. 10.1016/j.marpolbul.2006.03.014 16712877

[7] Laist DW, Knowlton AR, Mead JG, Collet AS, Podesta M. Collisions between ships and whales. Marine Mammal Science. 2001;17(1):35–75

[8] Van Waerebeek K, Baker AN, Felix F, Iniguez M, Sanino GP, Secchi E, et al Vessel collisions with small cetaceans worldwide and with large whales in the Southern Hemisphere: an initial assessment. Latin American Journal of Aquatic Mammals. 2007;6(1):43–69

[9] European Commission. Commission staff working paper: Executive summary of the impact assessment. European Commission, Brussels, Belgium [Internet]. 2013 Available at: http://ec.europa.eu/maritimeaffairs/policy/maritime_spatial_planning/documents/swd_2013_64_en.pdf.

[10] Hoyt E. Sustainable ecotourism on Atlantic islands, with special reference to whale watching, Marine Protected Areas and sanctuaries, for cetaceans. Biology & Environment: Proceedings of the Royal Irish Academy. 2005;105(3):141–154.

[11] Notarbartolo di Sciara G, Birkun Jr A. Conservation needs and strategies. In G. Notarbartolo di Sciara, ed. Cetaceans of the Mediterranean and Black Seas: State of knowledge and conservation strategies. A report to the ACCOBAMS Secretariat, Monaco, 2002 Feb; 21 pp.

[12] Dede A, Amaha Öztürk A, Tonay MA. Cetacean Surveys in the Istanbul (Bosphorus) Strait In 2006, 22nd Annual Conference of European Cetacean Society, Egmond aan Zee, Hollanda, 10–12 Mart 2008, 22:22.

[13] Baş AA, Öztürk AA, Öztürk Öztürk B.. Selection of critical habitats for bottlenose dolphins (Tursiops truncatus) based on behavioural data, in relation to marine traffic in the Istanbul Strait, Turkey. Marine Mammal Science. 2015;31:979–997.

[14] Nowacek SM, Wells RS, Solow AR. Short‐term effects of boat traffic on bottlenose dolphins, *Tursiops truncatus*, in Sarasota bay, Florida. Marine Mammal Science. 2001;17(4):673–688.

[15] Lusseau D. Effects of tour boats on the behaviour of bottlenose dolphins: using Markov chains to model anthropogenic impacts. Conservation Biology. 2003;17(6):1785–1793.

[16] Lusseau D. Residency pattern of bottlenose dolphins *Tursiops* spp. in Milford Sound, New Zealand, is related to boat traffic. Marine Ecology Progress Series. 2005;295:265–272.

[17] Lusseau D. The short-term behavioural reactions of bottlenose dolphins to interactions with boats in doubtful sound, New Zealand. Marine Mammal Science. 2006;22(4):802–818.

[18] Williams RM, Lusseau D, Hammond PS. Estimating relative energetic costs of human disturbance to killer whales (*Orcinus orca*). Biological Conservation. 2006;133(3):301–311.

[19] Gordon J, Leaper R., Hartley FG, Chappell O. Effects of whale-watching vessels on the surface and underwater acoustic behaviour of sperm whales off Kaikoura, New Zealand. Science and Research Series 52 1992.

[20] Barr K, Slooten E. Effects of tourism on dusky dolphins at Kaikoura. International Whaling Commission Scientific Committee. 1998. SC/50/WW10. 30 pp.

[21] Bejder L, Dawson SM, Harraway JA. Responses by Hector's dolphins to boats and swimmers in Porpoise Bay, New Zealand.” Marine Mammal Science. 1999;15(3):738–750.

[22] Van Parijs SM, Corkeron PJ. Boat traffic affects the acoustic behaviour of Pacific humpback dolphins, *Sousa chinensis*. Journal of the Marine Biological Association of the UK. 2001;81(03):533–538.

[23] Hastie GD, Wilson B, Tufft LH, Thompson PM. Bottlenose dolphins increase breathing synchrony in response to boat traffic. Marine Mammal Science. 2003;19(1):74–84

[24] Lemon M., Lynch TP, Cato DH, Harcourt RG. Response of travelling bottlenose dolphins (*Tursiops aduncus*) to experimental approaches by a powerboat in Jervis Bay, New South Wales, Australia. Biological Conservation. 2006;127(4):363–372.

[25] Stockin KA, Lusseau D, Binedell V, Wiseman N, Orams MB. Tourism affects the behavioural budget of the common dolphin *Delphinus* sp. in the Hauraki Gulf, New Zealand. Marine Ecology Progress Series. 2008;355:287–295.

[26] Christiansen F, Rasmussen MH, Lusseau D. Inferring activity budgets in wild animals to estimate the consequences of disturbances. Behavioural Ecology. 2013;24(6):1415–1425

[27] Christiansen F, Lusseau D. Linking behaviour to vital rates to measure the effects of non-lethal disturbance on wildlife. Conservation Letters. 2015;8(6):424–431.

[28] Bejder L, Samuels A, Whitehead H, Gales N. Interpreting short-term behavioural responses to disturbance within a longitudinal perspective. Animal Behaviour. 2006;72(5):1149–1158.

[29] Currey RJC, Dawson SM, Slooten E. An approach for regional threat assessment under IUCN red list criteria that is robust to uncertainty: the Fiordland bottlenose dolphins are critically endangered. Biological Conservation. 2009;142(8):1570–1579.

[30] Lusseau D. The hidden cost of tourism: detecting long-term effects of tourism using behavioural information. Ecology and Society. 2004;9(1):2.

[31] Christiansen F, Lusseau D. Understanding the ecological effects of whale watching on cetaceans Pages 177–192 in Higham J, Bejder L, Williams R, editors. Whale-watching, sustainable tourism and ecological management. Cambridge University Press, Cambridge, UK 2014.

[32] Anonymous. Annual report (in Turkish). T. C. Ulastirma, Denizcilik ve Haberlestirme Bakanlığı [Internet]. 2013. Available at: http://www.kiyiemniyeti.gov.tr.

[33] Istanbul Port Authority. Domestic marine traffic guide [Internet]. 2011. Available at: http://www.istanbullimangovtr/tr/indexphp#.

[34] Denizcilik Müsteşarlığı TC. Türk Boğazları Gemi Trafik Yönetim ve Bilgi Sistemi (GTYBS)—Gemi Trafik Hizmetleri (GTH) Projesi [Internet]. 2005. Available at: htpp://www.denizcilik.gov.tr Turkish.

[35] Simmonds M, Dolman S, Weilgart L. Oceans of noise: A WDCS science report. Whale and Dolphin Conservation Society, Plymouth, MA 2004.

[36] Anonymous. Identification and protection of special areas and particularly sensitive sea areas. IMO Assembly, 61th Session. MEPC. 2010. 61/9. p 14.

[37] Constantine R., Brunton DH, Dennis T. Dolphin-watching tour boats change bottlenose dolphin (*Tursiops truncatus*) behaviour. Biological Conservation. 2004;117(3):299–307.

[38] Neumann DR, Orams MB. Impacts of ecotourism on short-beaked common dolphins (*Delphinus delphis*) in Mercury Bay, New Zealand. Aquatic Mammals. 2006;32(1):1–9

[39] Meissner AM, Christiansen F, Martinez EM, Pawley MDM, Orams MB, Stockin KA. Behavioural effects of tourism on oceanic common dolphins, Delphinus sp., in New Zealand: the effects of Markov analysis variations and current tour operator compliance with regulations. PLos ONE 10 2015. 10.1371/journal.pone.0116962 PMC4286237 25565523

[40] Bejder L, Samuels A. Evaluating impacts of nature-based tourism on cetaceans Pages 229–256 in Gales N, Hindell M, Kirkwood R, editors. Marine Mammals: Fisheries, Tourism and Management Issues. Collingwood: CSIRO 2003.

[41] Lusseau D, Bain DE, Williams R, Smith JC. Vessel traffic disrupts the foraging behaviour of southern resident killer whales *Orcinus orca*. Endangered Species Research. 2009;6(3):211–221.

[42] Christiansen F, Lusseau D, Stensland E, Berrgren P. Effects of tourist boats on the behaviour of Indo-Pacific bottlenose dolphins off the south coast of Zanzibar. Endang Species Res. 2010;11:91–99.

[43] Fleiss JL. The measurement of interrater agreement. Statistical methods for rates and proportions. 1981;2:212–236.

[44] R Core Team. R: A language and environment for statistical computing. R Foundation for Statistical Computing, Vienna, Austria 2014 Available from http://www.r-project.org/

[45] Hildebrand J. Anthropogenic and natural sources of ambient noise in the ocean. Marine Ecological Progess Series. 2009;395:5–20

[46] McDonald MA, Hildebrand JA, Wiggins SM. Increases in deep ocean ambient noise in the Northeast Pacific west of San Nicolas Island, California. The Journal of the Acoustical Society of America. 2006;120:711–718. 16938959 10.1121/1.2216565

[47] Bain DE, Trites AW, Williams R. A model linking energetic effects of whale watching to killer whale (*Orcinus orca*) population dynamics. Friday Harbour Laboratories, University of Washington, Friday Harbour, Washington 2002.

[48] Bain DE, Smith JC, Williams R, Lusseau D. Effects of vessels on behaviour of Southern Resident killer whales (Orcinus spp.) 2003–2005. NMFS Contract report No. AB133F05SE3965. National Marine Fisheries Service, National Marine Mammal Laboratory, Seattle, Washington. 2006.

[49] Lusseau D, Slooten L, Currey RJ. Unsustainable dolphin-watching tourism in Fiordland, New Zealand”. Tourism in Marine Environments. 2006;3(2):173–178.

[50] Polacheck T, Thorpe L. The swimming direction of harbour porpoise in relationship to a survey vessel. Report of the International Whaling Commission. 1990;40:463–470.

[51] Dyndo M, Wisniewska DM, Rojano-Donate L, Madsen PT. Harbour porpoises react to low levels of high frequency vessel noise. Scientific reports. 2015;5. 10.1038/srep11083 PMC4476045 26095689

[52] Kastelein RA, Steen N, De Jong C, Wensveen PJ, Verboom WC. Effect of broadband-noise masking on the behavioural response of a harbour porpoise (*Phocoena phocoena*) to 1-s duration 6–7 kHz sonar up-sweeps. The Journal of the Acoustical Society of America. 2011;129(4):2307–2315 10.1121/1.3559679 21476686

[53] Hermannsen L, Beedholm K, Tourgaard J, Madsen PT. High frequency components of ship noise in shallow water with a discussion of implications for harbour porpoises (*Phocoena phocoena*). The Journal of the Acoustical Society of America. 2014;136(4):1640–1653. 10.1121/1.4893908 25324068

[54] Dans SL, Crespo EA, Pedraza SN, Degrati M, Garaffo GV. Dusky dolphin and tourist interaction: effect on diurnal feeding behaviour. Marine Ecology Progress Series. 2008;369:287–296.

[55] Bryant PJ, Lafferty CM, Lafferty SK. Reoccupation of Laguna Guerrero Negro, Baja California, Mexico, by gray whales Pages 375–387 in Jones ML, Swartz SL, Leatherwood S, editors. The Gray whale (*Eschrichtius robustus*). Academic Press, Orlando, FL, USA 1984.

[56] Richardson WJ, Finley KJ, Miller GW, Davis RA, Koski WR. Feeding, social and migration behaviour of bowhead whales, *Balaena mysticetus*, in Baffin Bay vs. the Beaufort Sea—regions with different amounts of human activity. Marine Mammal Science. 1995;11(1):1–45.

[57] Notarbartolo di Sciara G. The Ligurian Sea international sanctuary for Mediterranean cetaceans: rationale, history and current status. Proceedings of the workshop Protected Areas for Cetaceans. European Cetaceans Newsletter. 2001;38:28–30.

[58] Aguilar de Soto N, Johnson M, Madsen PT, Tyack PL, Bocconcelli A, Borsani, JF. Does intense ship noise disrupt foraging in deep-diving Cuvier’s beaked whales (*Ziphius cavirostris*). Marine Mammal Science. 2006;22(3):690–699.

[59] Martin J, Sabatier Q, Gowan TA, Giraud C, Gurarie E, Calleson CS, Ortega-Ortiz JG, Deutsch CJ, Rycyk A, Koslovsky SM. A Quantitative Framework for Investigating Risk of Deadly Collisions between Marine Wildlife and Boats. Methods in Ecology and Evolution.2015.

[60] Dede A, Öztürk AA, Akamatsu T, Tonay AM, Öztürk B. Long-term passive acoustic monitoring revealed seasonal and diel patterns of cetacean presence in the Istanbul Strait, Journal of the Marine Biological Association of the United Kingdom. 2013 1–8.

[61] Bejder L, Samuels A, Whitehead H, Gales N, Mann J, Connor R, Heithaus M, Watson-Capps J, Flaherty C, Kruetzen M. Decline in relative abundance of bottlenose dolphins exposed to long‐term disturbance. Conservation Biology. 2006: 20(6), 1791–1798. 10.1111/j.1523-1739.2006.00540.x 17181814

[62] Öztürk B, Öztürk AA. On the biology of the Turkish Straits System. Bulletin de Institute Oceanographique, Monaco CIESM Science Series No 2 1996 pp 205–221.

