# Correction: The effects of marine traffic on the behaviour of Black Sea harbour porpoises (*Phocoena phocoena relicta*) within the Istanbul Strait, Turkey

**DOI:** 10.1371/journal.pone.0183597

**Published:** 2017-08-16

**Authors:** Aylin Akkaya Bas, Fredrik Christiansen, Ayaka Amaha Öztürk, Bayram Öztürk, Caley McIntosh

The following information is missing from the Funding section: This work has been partly funded by the EC FP7 PERSEUS Project (Grant. Agr. 287600) and by the Scientific Research Projects Coordination Unit of Istanbul University (Project number FOA-2016-20530).
